# Clinical and cognitive correlates tractography analysis in patients with white matter hyperintensity of vascular origin

**DOI:** 10.3389/fnins.2023.1187979

**Published:** 2023-06-16

**Authors:** Qinmei Kuang, Muhua Huang, Yumeng Lei, Lin Wu, Chen Jin, Jiankun Dai, Fuqing Zhou

**Affiliations:** ^1^Department of Radiology, First Affiliated Hospital of Nanchang University, Nanchang, Jiangxi, China; ^2^Clinical Research Center for Medical Imaging in Jiangxi Province, Nanchang, China; ^3^Department of Radiology, Nanchang First Hospital, Nanchang, Jiangxi, China; ^4^GE Healthcare, MR Research China, Beijing, China

**Keywords:** white matter hyperintensity, diffusion magnetic resonance imaging, hypertension, cognition, body mass index, tractography

## Abstract

**Purpose:**

White matter hyperintensity lesions (WMHL) in the brain are a consequence of cerebral small vessel disease and microstructural damage. Patients with WMHL have diverse clinical features, and hypertension, advanced age, obesity, and cognitive decline are often observed. However, whether these clinical features are linked to interrupted structural connectivity in the brain requires further investigation. This study therefore explores the white matter pathways associated with WMHL, with the objective of identifying neural correlates for clinical features in patients with WMHL.

**Methods:**

Diffusion magnetic resonance imaging (MRI) and several clinical features (MoCA scores, hypertension scores, body mass index (BMI), duration of hypertension, total white matter lesion loads, and education.) highly related to WMHL were obtained in 16 patients with WMHL and 20 health controls. We used diffusion MRI connectometry to explore the relationship between clinical features and specific white matter tracts using DSI software.

**Results:**

The results showed that the anterior splenium of the corpus callosum, the inferior longitudinal fasciculus, the anterior corpus callosum and the middle cerebellar peduncle were significantly correlated with hypertension scores (false discovery rate (FDR) = 0.044). The anterior splenium of the corpus callosum, the left thalamoparietal tract, the inferior longitudinal fasciculus, and the left cerebellar were significantly correlated with MoCA scores (FDR = 0.016). The anterior splenium of corpus callosum, inferior fronto-occipital fasciculus, cingulum fasciculus, and fornix/fimbria were significantly correlated with body mass index (FDR = 0.001).

**Conclusion:**

Our findings show that hypertension score, MoCA score, and BMI are important clinical features in patients with WMHL, hypertension degree and higher BMI are associated with whiter matter local disconnection in patients with WMHL, and may contribute to understanding the cognitive impairments observed in patients with WMHL.

## Introduction

White matter hyperintensities lesions (WMHL) are characterized by multiple punctate, patchy, or confluent hyperintensities in the bilateral periventricular or subcortical white matter on T2-weighted images or T2-weighted fluid-attenuated inversion recovery images (T2-Flair) and are one of the common imaging markers of cerebral small vessel disease (CSVD) (Wardlaw et al., 2013). Various factors contribute to WMHL formation, including age, female sex, hypertension, hyperlipidemia, apolipoprotein Eε4 allele, diabetes, smoking, and alcohol consumption (Alber et al., 2019; Frey et al., 2019). Previous studies have demonstrated that WMHL are associated with an increased risk of stroke, cognitive decline, dementia, and death (Debette and Markus, 2010). Even in healthy aging, higher whole-brain lesion volume and regional WMHL load are consistently associated with poorer cognitive performance in processing speed, memory, and executive functioning (van Rooden et al., 2018).

Previous studies have shown anomalies not only within T2-WMHL but also in apparently “normal appearing white matter (NAWM)” (Maillard et al., 2011). Over time, abnormal changes in NAWM precede WMHL progression, known as WMHL penumbra (Wu et al., 2019). Cognitive impairment in patients with WMHL is correlated with microstructural destruction of various white matter fibers, which may include “disconnection” of cortical–subcortical pathways (Yuan et al., 2017; Lu et al., 2021), and the relationship between overall cognitive function and white matter integrity may be closer than that with blood supply (Zhong et al., 2017).

Serious vascular diseases can lead to cortical and subcortical infarction, affect brain functional areas, and result in vascular cognitive impairment (Frantellizzi et al., 2020; Meng et al., 2022). Compared to all other regions, the front permanent WMHL has more pronounced diffusion tensor imaging metrics changes with the same Fazekas grades, which may represent the effects of severe demyelination within the frontal periventricular WMHL (Min et al., 2021). The results also indicate an increased WMHL burden selectively in deep white matter in obese subjects with high visceral fat accumulation. Independent of common obesity comorbidities such as hypertension, it may be that the visceral obesity contributes to deep white matter lesions through increases in proinflammatory cytokines (Min et al., 2021). In fact, most patients with WMHL may not lead to typical clinical symptoms because of collateral circulation development in these ischemic regions.

However, consensus is lacking as to regarding which etiology or clinical characteristic and corresponding white matter tract provide the most accurate reflection of patients with WMHL and may be used as neural correlates (white matter fiber bundle segments correlated with study variables) to assist clinical diagnosis. We hypothesized that even asymptomatic patients with WMHL may have microstructural impairment. This study intends to use the diffusion MRI connectome analysis method to identify neural correlates that reflect the underlying deficits in WMHL and to identify the most representative neural correlates for WMHL.

## Materials and methods

### Subjects

All subjects provided signed, informed consent prior to inclusion in the study in accordance with the local institutional review board. From May 2020 to December 2021, patients with WHM (*n* = 16) from inpatient were recruited to the first affiliated hospital of Nanchang University. The inclusion criteria for WMHL subjects were as follows: (1) presence of grade 2 or 3 WMHL according to Fazekas scale on FLAIR; (2) no contraindications to MRI. Exclusion criteria were as follows: (1) history of ischemic stroke with infarct size greater than 1.5 cm in diameter or cardiogenic cerebral embolism, (2) cerebral hemorrhage, (3) internal carotid artery or vertebral artery stenosis (>50%) or coronary atherosclerosis heart disease, (4) WMHL due to immune-mediated inflammatory demyelinating disease (multiple sclerosis, neuromyelitis optica, acute disseminated encephalomyelitis), metabolic leukodystrophy and genetic leukoencephalopathy, and (5) other neurological disorders.

Healthy controls (HC, *n* = 20) were randomly recruited, who had no history of hypertensive disease, traumatic brain injurie, neurologic diseases, or brain abnormalities based on conventional MRI (T1 weight image, T2 weight image, T2 fluid attenuated inversion recovery).

### Image acquisition

A total of 36 diffusion MRI scans were included in the connectometry database. The diffusion images were obtained on a 3 T MR system (Premier, GE Healthcare, Madison, Wisconsin) using a 2D EPI diffusion sequence. Foam padding was used to position subjects on the coil so that the cervical spine was straight but comfortable to minimize head motion. The spatial resolution was 0.875 mm × 0.875 mm × 2.5 mm isotropic. TR = 5,500 ms, TE =71 ms. FOV 224 mm × 224 mm. The diffffusion data was acquired with 100 directions with b-values (3,000 s/mm^2^). Thickness of the slice was 2 mm. Axial conventional T1WI, T2WI, T2-FLAIR (fluid-attenuated inversion recovery) were acquired in the brain for each patient’s diagnosis. The following scan parameters were used: axial T1WI: TR = 250 msec, TE = 2.46 msec, slice number = 19, slice thickness = 5.0 mm, FOV = 24 × 24 cm, matrix size = 256 × 256; axial T2WI: repetition time (TR) = 4,000 msec, echo time (TE) = 113 msec, slice number = 19, slice thickness = 5.0 mm, field of view (FOV) = 24 × 24 cm, matrix size = 256 × 256; axial T2-FLAIR: TR =7,000 msec, TE = 162 msec, slice number = 19, slice thickness = 5.0 mm, FOV = 24 × 24 cm, and matrix size = 256 × 256.

### Lesion load, hypertension scores assessments

A binary lesion mask was manually drawn from T2WI to identify all visible lesions by an experienced neuroradiologist (F.Z.) using MRIcron. The T2WI was coregistered with the T1-weighted structural image. After co-registration with the T1WI-based individual brain according to the Montreal Neurological Institute (MNI) standard brain dimensions, and this information was used to warp the lesion mask [as the normalized total white matter lesion load (TWMLL)] reflected the TWMLL relative to the standard MNI brain volume rather than the individual brain volume so that the effects of differences in brain volume were controlled (Pelletier et al., 2004).

According to the diagnosis of hypertension and the cardiovascular risk factors stratification standard (Williams et al., 2018), hypertension is divided into Grade I, Grade II, and Grade III, and we give corresponding scores of 1, 2 and 3; the degree of danger includes low risk, medium risk, high risk and very high risk, and the corresponding scores are 1, 2, 3, and 4. Total hypertension score is equal to hypertension level score plus hypertension risk score. Based on patient admission diagnosis, the score of the WMHL patient is 2–5, as shown in Table 1.

**Table 1:** Demographic and clinical characteristics of the study population.

	Patients with WMHL (*n* = 16)	HC (*n* = 20)	*p* value
Age (years)	57.06 ± 6.85	55.90 ± 5.39	0.31^a^
Sex (female: male)	8:8	12:8	0.46^b^
Education level (years)	8.43(5–16)	9.4(5–14)	0.57^a^
Duration (years)	8 (2–20)	–	–
MoCA scores	20.44 ± 4.40	24.60 ± 2.01	0.000^a^
TWMML(cm^3^)	67.62(19.56–285.69)	–	–
Hypertension scores	3.06 ± 1.29	–	–
BMI(kg/m^2^)	23.43 ± 3.47	21.31 ± 3.16	0.003^a^

MoCA, Montreal Cognitive Assessment; BMI, body mass index; WMHL, White matter hyperintensity lesions; HC, Healthy controls; TWMLL, total white matter lesion loads in the normalized standard brain. a: Two-sample *t* test; b: Chi square test.

### Demographic, BMI, clinical, and neuropsychological assessments

Demographic, clinical, and neuropsychological characteristics, including age, sex, disease duration (From the first diagnosis of the disease to enrollment), height (m) and weight (kg), Montreal Cognitive Assessment (MoCA). body mass index (BMI) is a person’s weight in kilograms divided by the square of height in meters (kg/m^2^).

### Diffusion MRI connectometry analysis

The diffusion data were reconstructed in the MNI space using q-space diffeomorphic reconstruction (Yeh and Tseng, 2011) to obtain the spin distribution function (SDF) (Yeh et al., 2010) using DSI Studio.^1^ With the default settings, a diffusion sampling length ratio of 1.25 was used. The sdf values were used in the connectometry analysis.

Diffusion MRI connectometry analysis enabled us to further investigate the specific pathways associated with study variable (Yeh et al., 2016). In this study, our study variables included hypertension scores, MoCA scores, BMI, TWMLL, duration of hypertension, and education. Because most study variable were highly correlated with each other, we first conducted a principal component analysis (PCA) to isolate the principal components that explained overall score variability. This avoided overfitting in the regression model. A multiple regression model was used to adjust for sex and age, and the following default parameters were used. At the group level, we used concatenated HC and WMHL data to create a connectometry database. Considering of higher length thresholds provide more specific results for identifying affected tracks, while lower length thresholds are more sensitive to potentially affected tracks. In our study, different length thresholds (30 mm, 40 mm, and 50 mm) were used to study the correlation at different significance levels between the two groups. To further analyze the study variables associated with tracks, we only used WMHL group data to create a connectometry database, a 40 mm length threshold was used to select tracks. The seeding density was 20 seeds per mm^3^. Local connectomes were tracked using a deterministic fiber tracking algorithm. Track trimming was performed with 2 iterations. All tracks generated from bootstrap resampling were included. To estimate the false discovery rate (FDR), 2000 random permutations were applied to the group label to obtain the null distribution of the track length. An FDR lower than 0.05 can be considered as significant.

## Results

### Demographic and clinical data

Sixteen patients with WMHL and 20 HC were included in this study. Population and clinical characteristics are summarized in Table 1. The WMHL has significantly lower MOCA scores and higher BMI than HC (respective *p* = 0.000 and *p* = 0.003).

### Principle component analysis

The PCA indicated that only three components were sufficient to explain 87.196% of the score variance. The first component was hypertension scores, with a coefficient of 0.712, which explained 32.936% of the behavioral data. The second component was MoCA, with a coefficient of 0.812, which explained 26.542% of the behavioral data. The third component was BMI, with a coefficient of 0.825, which explained the other 27.718%. The results are shown in Table 2.

**Table 2:** Principle component analysis of the study variables.

	First component (IC1)	Second component (IC2)	Third component (IC3)
Variance explained(%)	32.939	26.542	27.718
	Component coefficient	Component coefficient	Component coefficient
Hypertension scores	0.712	0.511	−0.236
MoCA scores	0.466	0.812	0.439
BMI	0.374	−0.231	0.825
nTWMLL	−0.613	0.468	0.165
Duration	0.027	−0.774	0.153
Education	−0.645	0.292	0.289

### Tracks correlated with group

**Figure 1:**
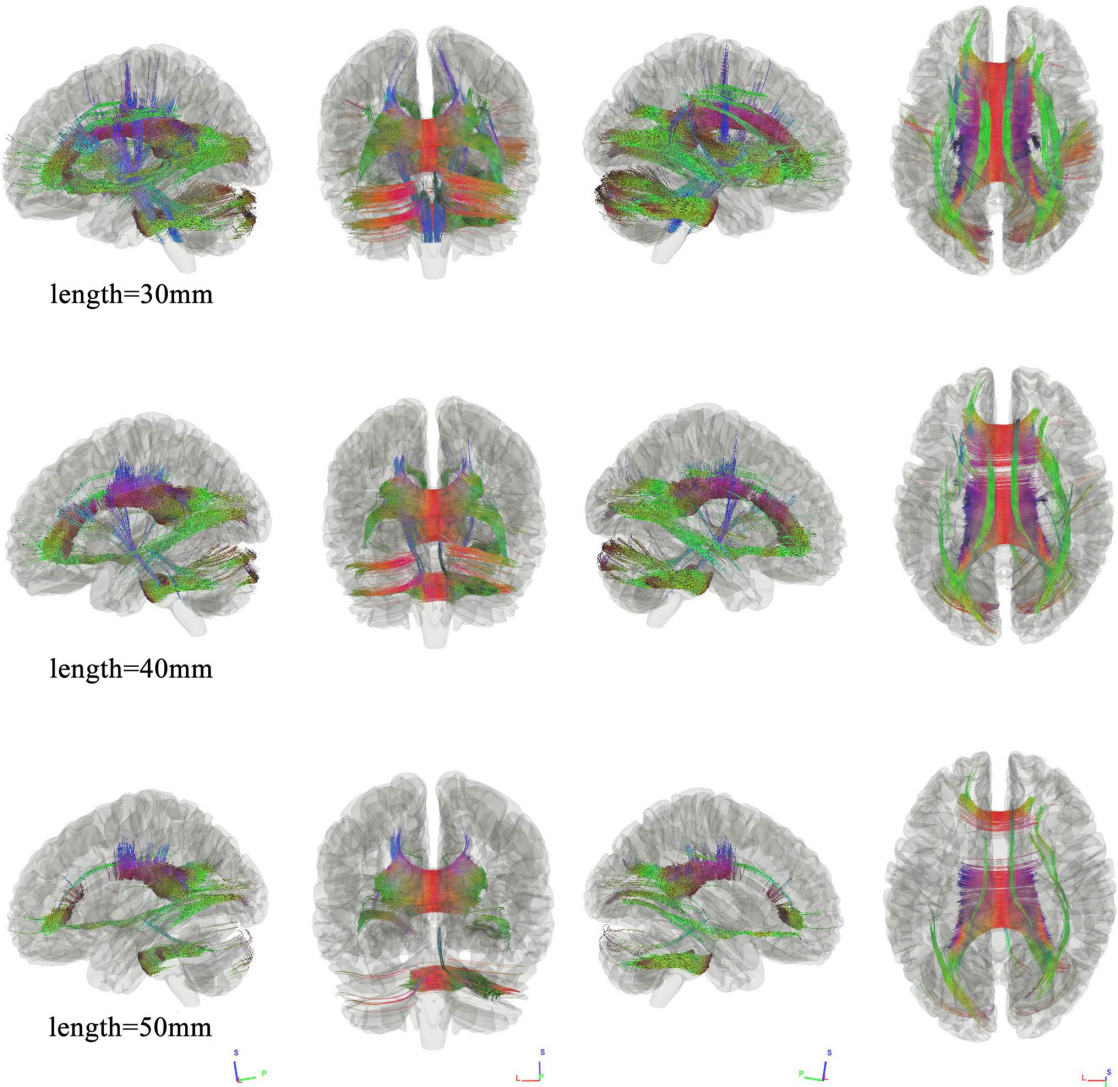
Connectometry results under different length thresholds between the two groups. Connectometry analysis identified connectivity differences that appeared and wide-spread the whole brain, involving bilateral association bundles, corpus callosum, projection bundles, and cerebellar peduncle. (FDR was 0.023, 0.013, 0.020 when the length was 30 mm, 40 mm, 50 mm, respectively).

We use different length thresholds (30 mm, 40 mm, and 50 mm) to study the correlation at different significance levels between the two groups. Figure 1 shows the WMHL group had decreased fiber connectivity in the brain network compared with the HC. The FDR was 0.023, 0.013, 0.020 when the length was 30 mm, 40 mm, 50 mm, respectively. The analysis showed that the differential tractography involving bilateral association bundles, corpus callosum, projection bundles and cerebellar peduncle.

### Tracks correlated with hypertension scores

**Figure 2:**
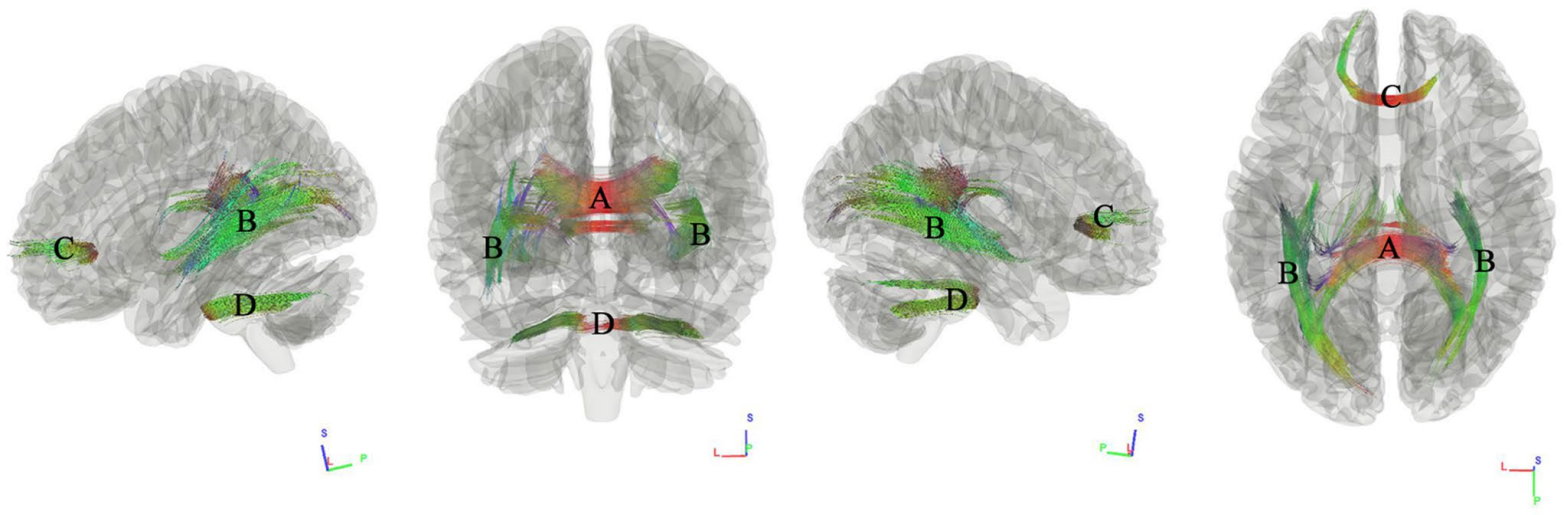
Tracks negatively correlated with hypertension scores in patients with WMHL. Connectometry analysis identified **(A)** anterior splenium of corpus callosum, **(B)** inferior longitudinal fasciculus, **(C)** anterior corpus callosum, **(D)** middle cerebellar peduncle with decreased connectivity to hypertension cores (FDR = 0.044).

We first used connectometry analysis to map the fiber pathways correlated with the first component of PCA, which was predominantly weighted by the hypertension score. Figure 2 shows the tracks negatively correlated with hypertension scores. The FDR was 0.044. The analysis indicated that the pathways correlated with hypertension cores were anterior splenium of corpus callosum (ASCC), bilateral inferior longitudinal fasciculus(ILF), anterior corpus callosum (ACC), middle cerebellar peduncle.

### Tracks correlated with MoCA

**Figure 3:**
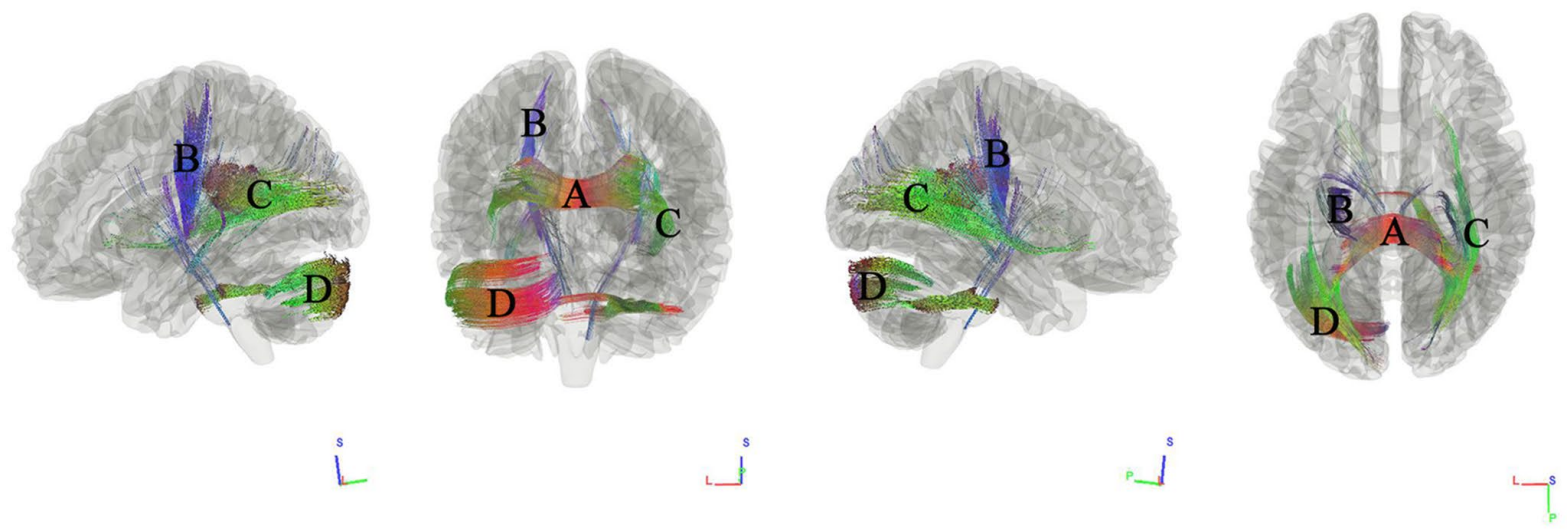
Tracks negatively correlated with MoCA scores in patients with WMHL. Connectometry analysis identified **(A)** anterior splenium of the corpus callosum, **(B)** left thalamoparietal pathways, **(C)** right inferior longitudinal fasciculus, and **(D)** left cerebellar with decreased connectivity to MoCA scores (FDR = 0.016).

We used connectometry analysis to map fiber pathways correlated with the second PCA component, which was predominantly weighted by the MoCA scores. Figure 3 shows the tracks negatively correlated with MoCA scores. The FDR was 0.016. The analysis indicated that the pathways correlated with MoCA scores were ASCC, left thalamoparietal pathways, right inferior longitudinal fasciculus ILF, and left cerebellar.

### Tracks correlated with BMI

**Figure 4:**
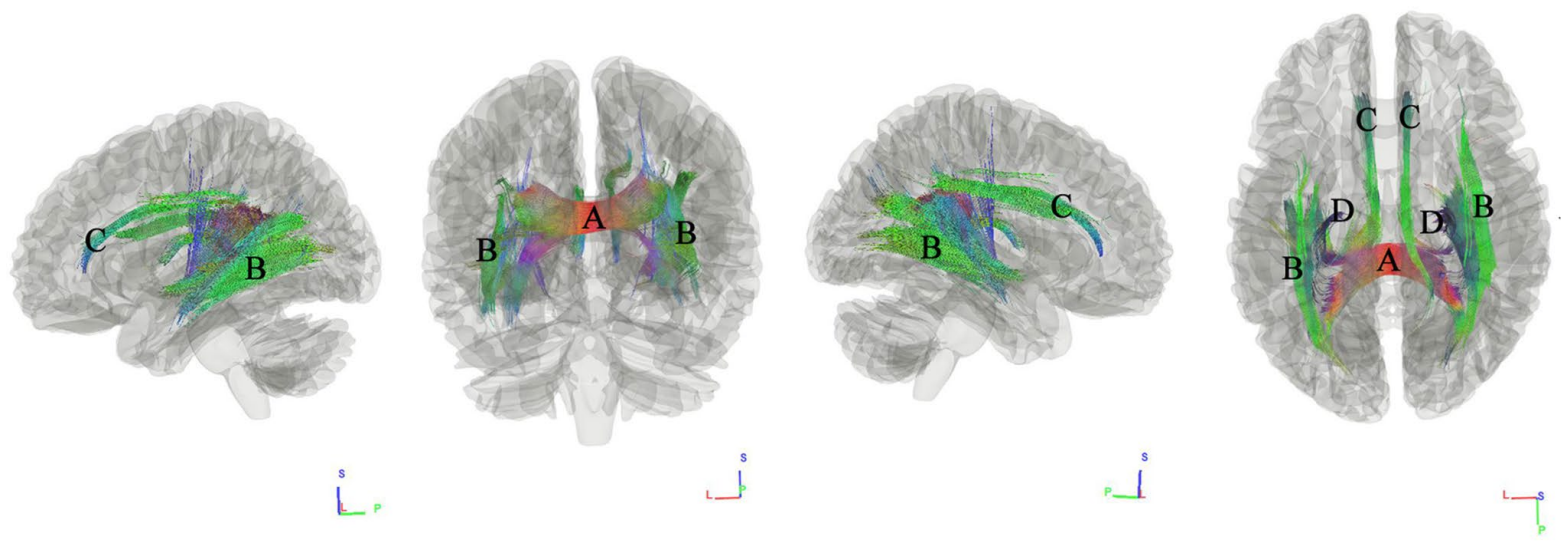
Tracks negatively correlated with BMI in patients with WMHL. Connectometry analysis identified **(A)** the anterior splenium of the corpus callosum, **(B)** inferior fronto-occipital fasciculus, **(C)** cingulum fasciculus, and **(D)** fornix/fimbria with decreased connectivity to BMI (FDR = 0.001).

We used connectometry analysis to map the fiber pathways correlated with the third PCA component, which was predominantly weighted by the BMI. Figure 4 shows the tracks negatively correlated with BMI. The FDR was 0.001. The analysis indicated that the pathways correlated with BMI were ASCC, bilateral inferior fronto-occipital fasciculus (IFOF), bilateral cingulum fasciculus, fornix/fimbria.

## Discussion

This study provides evidence for the association between white matter fibers and hypertension scores, MoCA scores, and BMI in patients with WMHL. Anatomically, ASCC, bilateral ILF, ACC, and middle cerebellar peduncle were significantly correlated with hypertension scores. ASCC, left thalamoparietal pathways, right ILF, and left cerebellar were significantly correlated with MoCA scores. ASCC, IFOF, cingulum fasciculus, fornix/fimbria were significantly correlated with BMI.

Hypertension is a well-known significant risk factor for WMHL, which is closely related to WMHL progression. Among hypertensive individuals, the prevalence of WMHL may rise to 40–44% (Liao et al., 1996; Verhaaren et al., 2013; Gebeily et al., 2014; Sargurupremraj et al., 2020). In addition, hypertension is highly relevant to the annual progression of WMHL. People with uncontrolled untreated hypertension had significantly more WMHL progression than regular treatment (Verhaaren et al., 2013; Shen et al., 2022). In our study, we found that decreased connectivity in ASCC, bilateral ILF, ACC, and middle cerebellar peduncle were significantly negatively correlated with hypertension scores. Previous studies have reported the relationship between hypertension and lower microstructural fiber density, macrostructural fiber bundle cross-section, and a combination of both, such as corpus callosum, ILF, and middle cerebellar peduncle (Gons et al., 2012; Li et al., 2015; Wong et al., 2017; Andica et al., 2022). Studies have also found impaired microstructural integrity of the corpus callosum in hypertensive patients compared to normotensive patients in individuals with cerebral small vessel disease (Gons et al., 2012). This may be related to the vulnerability of posterior brain regions to vascular risk factors (Artero et al., 2004). The fibers in splenium are projections from the occipital-parietal and temporal cortex. It has been shown to be involved in language, reading, and calculation skills, IQ, conduct, and consciousness (Blaauw and Meiners, 2020). The ILF is a long-range white matter pathway that primarily connects the occipital lobe of the brain with the anterior temporal lobe. It may involve a wide range of brain functions concerning the visual modality, including object, face and place processing, reading, lexical and semantic processing, emotion processing, and visual memory (Herbet et al., 2018). Previous studies have mostly found that hypertension shows significantly reduced white matter integrity in the bilateral superior longitudinal fasciculus (Li et al., 2015). In patients with WMHL with higher FA values, the integrity of the ILF moderates the association between higher WMHL and slower gait speed (Rosario et al., 2016). The middle cerebellar peduncles are the main afferent pathway to the cerebellum and are composed of white matter fibers originating from the contralateral pontine nuclei. The pontine nuclei are intermediate gray matter scattered in the basis pons and part of the corticopontocerebellar pathway that controls not only the action of motor tasks but also the planning and initiation of movements. Thus, difficulty walking (cerebellar ataxia), difficulty speaking (scanning speech), and in some cases vertigo and facial weakness are common clinical manifestations of middle cerebellar peduncles lesion (Morales and Tomsick, 2015).

Compared to HC, patients with WMHL has been associated with an increased risk of cognitive impairment and a significantly greater burden (Hu et al., 2021; Dadar et al., 2022). Our study found that hypertension scores and MoCA scores show a significant correlation with decreased connectivity in corpus callosum and ILF. Previous studies have reported that disruption of the corpus callosum microstructure especially the WMHL penumbra of the corpus callosum body may contribute to cognitive deficits associated with subcortical ischemic vascular disease (Qiu et al., 2021). Microstructural disruption of the right IFOF and ILF contributes to WMHL-related cognitive impairment (Chen et al., 2020) which is basically consistent with our research results, the effect of WMHL on the microstructural integrity of WM tracts may propagate along tracts to distal regions beyond the penumbra (Liu et al., 2021), In addition, in our study, decreased connectivity in left thalamoparietal pathways and left cerebellar were negatively correlated with MoCA scores. The thalamus is a crucial node in networks supporting cognitive functions known to deteriorate with normal aging, including component processes of memory and executive functions of attention and information processing (Fama and Sullivan, 2015). The cerebellum has both sensorimotor and cognitive effects. The lesions of the anterior lobe lead to dyskinesia, and the lesions of the posterior lobe result in cerebellar cognitive affective syndrome, which may explain WMHL susceptibility to motor and emotional disorders or other symptoms (Schmahmann, 2019).

In our study, patients with WMHL had higher BMI. Interestingly, previous studies have reported that higher BMI contributed to an increased deep-periventricular WMHL ratio (Lampe et al., 2019), which may be due to obesity leading to higher white matter burden through inflammatory processes, as indexed by elevated IL-6. Negative BMI associations are widely distributed across white matter pathways in a largely bilateral pattern primarily in corpus callosum, which is consistent with our findings (Karlsson et al., 2013; Verstynen et al., 2013). In particular, we found that BMI was negatively correlated with cingulum fasciculus, fornix/fimbria, which are the most prominent tracts within the limbic system. The fornix is a C-shaped fiber bundle that provides strong connections from the hippocampus to other brain regions. The cingulum is another C-shaped structure of WM fiber wrapped around the frontal and temporal lobe above the corpus callosum. The anterior part is important for emotion processing, while the posterior region is involved in cognition. This result may show BMI’s heterogeneous association with white matter pathways across the brain (Verstynen et al., 2013).

Our research still has several limitations. First, the age range of patients and sample sizes were small, a larger sample should be used in future studies. Secondly, the lack of longitudinal traces cannot provide evidence for progressive changes between white matter hyperintensities and the main clinical features, in furture, research based on longitudinal data may make the correlations we observe more specific. In addition to the clinical features discussed in this paper, other factors such as smoking, alcohol consumption, diabetes, and hyperlipemia are risk factors for WMHL. Further research is needed to include hypertensive patients with different accompanying symptoms (eg. cerebrovascular disease, cardiovascular disease, kidney disease, etc.) in the future. Especially longitudinal studies may better describe this characteristic in patients with WMHL.

In conclusion, this study employed a novel approach to diffusion connectometry to identify hypertension related clinical features and corresponding white matter structures. This preliminary study results support hypertension degree and higher BMI are associated with white matter local disconnection in patients with WMHL, and may contribute to understanding the cognitive impairments observed in patients with WMHL.

## Data availability statement

The raw data supporting the conclusions of this article will be made available by the authors, without undue reservation.

## Ethics statement

The studies involving human participants were reviewed and approved by Medical Research Ethics Committee of the First Affiliated Hospital of Nanchang University. The patients/participants provided their written informed consent to participate in this study.

## Author contributions

FZ, QK, MH, YL, and LW: conceptualization. YL, QK, and MH: data collection. FZ, MH, QK, and LW: data analysis. FZ: funding analysis. MH, QK, FZ, LW, JD, and YL: investigation, writing—original draft, and writing—review and editing. FZ, LW, and YL: supervision. All authors contributed to the article and approved the submitted version.

## Funding

This study was supported by the National Science Foundation of China (Grant no 82160331).

## Conflict of interest

The authors declare that the research was conducted in the absence of any commercial or financial relationships that could be construed as a potential conflict of interest.

## Publisher’s note

All claims expressed in this article are solely those of the authors and do not necessarily represent those of their affiliated organizations, or those of the publisher, the editors and the reviewers. Any product that may be evaluated in this article, or claim that may be made by its manufacturer, is not guaranteed or endorsed by the publisher.

## Footnotes

^1^ http://dsi-studio.labsolver.org/
